# *Mycobacterium* *lepromatosis* Lepromatous Leprosy in US Citizen Who Traveled to Disease-Endemic Areas

**DOI:** 10.3201/eid2311.171104

**Published:** 2017-11

**Authors:** Abinash Virk, Bobbi Pritt, Robin Patel, James R. Uhl, Spencer A. Bezalel, Lawrence E. Gibson, Barbara M. Stryjewska, Margot S. Peters

**Affiliations:** Mayo Clinic, Rochester, Minnesota, USA (A. Virk, B. Pritt, R. Patel, J.R. Uhl, S.A. Bezalel, L.E. Gibson, M.S. Peters);; National Hansen’s Disease Programs, Baton Rouge, Louisiana, USA (B.M. Stryjewska)

**Keywords:** 16S ribosomal RNA gene PCR, lepromatosis, lepromatous, leprae, leprosy, tuberculosis and other mycobacteria, *Mycobacterium*, neuropathy, rheumatoid arthritis, United States, Mexico, travel, tourists, zoonoses, vector-borne infections

## Abstract

We report *Mycobacterium lepromatosis* infection in a US-born person with an extensive international travel history. Clinical symptoms, histopathology, and management are similar to those of infections caused by *M. leprae*. Clinicians should consider this pathogen in the diagnosis of patients with symptoms of leprosy who have traveled to endemic areas.

A 59-year-old white man born in the United States came to the Travel and Tropical Medicine Clinic at Mayo Clinic (Rochester, Minnesota, USA) in March 2017 reporting 12 months of progressive skin lesions and prior onset of peripheral neuropathy and arthritis. The lesions started on his forearms, then progressed to nearly diffuse involvement of his face, neck, ears, and trunk. The lesions were not pruritic, painful, or ulcerative. Two years earlier, arthralgia of his hips, knees, and hands led to a diagnosis of rheumatoid arthritis on the basis of positive results for rheumatoid factor, negative cyclic citrullinated peptide antibody, and antinuclear antibodies. He received prednisone for several weeks, then methotrexate (20 mg/wk). He also had an electromyography-confirmed sensorimotor peripheral neuropathy that began 1 year before this presentation. After an unsuccessful empiric course of topical corticosteroid therapy, skin biopsies were collected from his neck and forearm; results showed sheets of histiocytes and scattered well-formed granulomas with perineural involvement. Fite stain was positive for numerous intracellular acid-fast bacilli, leading to a diagnosis of lepromatous leprosy; the patient was referred to the Division of Infectious Diseases at Mayo Clinic for further evaluation.

The patient was an administrator who worked indoors and did not have substantive outdoor exposure. He was born and raised in the United States but had an extensive travel history as an adult to many countries over several decades, including 2 trips to the Pacific coast of Mexico (7 days each in Puerto Vallarta, Jalisco, in April 2005, and Acapulco in March 2007), a region to which *Mycobacterium lepromatosis* leprosy is endemic (*1*). He had no known exposure to a person with leprosy or to armadillos, which are known vectors for leprosy.

On physical examination, we found nearly diffuse erythema and induration of the patient’s face, ears, neck (Figure 1), and chest, as well as his upper extremities, more focally involving the dorsal aspects of the forearms. He had partial loss of eyebrows bilaterally. The right auricle had a small, crusted ulcer. His nasal mucosa was thickened and nasal passages almost blocked. Ulnar and superficial cervical nerves were not easily palpated. He showed no clinical signs of active synovitis.

**Figure 1:**
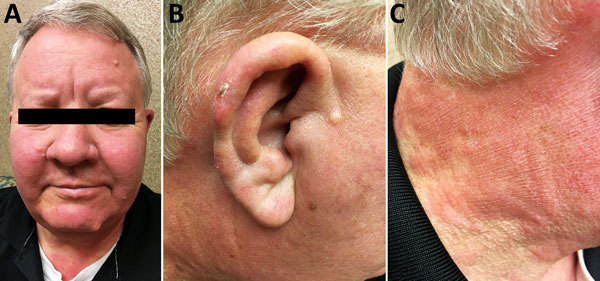
Signs of *Mycobacterium* *lepromatosis* infection in 59-year-old white male US citizen, 2017. A) *Leonine* *facies* with partial loss of eyebrows and nodular lesion of chin. B) Right ear nodularity with focal crusted ulceration. C) Confluent erythema from face to neck.

Initial laboratory test results were within reference ranges or negative, including complete blood count, erythrocyte sedimentation rate, C-reactive protein, creatinine, QuantiFERON-TB Gold In-Tube Test (Quest Diagnostics, Madison, NJ, USA), HIV serologies, rhumatoid factor (despite positive test 2 years prior), and cyclic citrullinated peptide antibodies. Chest radiograph results were unremarkable.

Skin biopsies of the neck, chin, and forearm showed patchy or diffuse granulomatous dermal inflammation, with foamy and epithelioid histiocytes. We found numerous acid-fast bacilli within histiocytes and invading nerves (Figure 2), highlighted by Fite, Gomori methenamine silver, and Gram stains; Ziehl-Neelsen stain highlighted only a few organisms. The combination of clinical, histopathologic, and histochemical staining features was diagnostic for multibacillary lepromatous leprosy.

**Figure 2:**
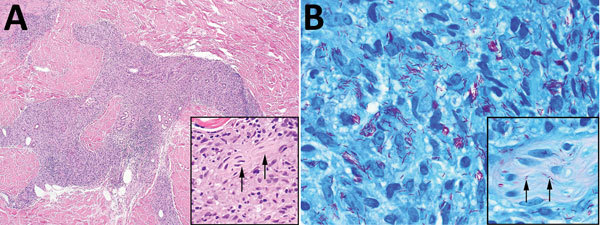
Skin biopsies of 59-year-old white male US citizen showing *Mycobacterium* *lepromatosis* infection, 2017. A) Hematoxylin and eosin–stained section of a specimen from the chin showing granulomatous dermal inflammation (original magnification ×100); inset shows nerve involvement (arrows) that is diagnostic for leprosy (original magnification ×400). B) Fite-stained section of a specimen from the chin highlights numerous acid-fast bacilli within histiocytes (original magnification ×1,000); inset shows peripheral nerve involvement (arrows) that is diagnostic for leprosy (original magnification ×1,000).

We achieved a diagnosis by using the broad-range 16S ribosomal RNA gene PCR assay on the formalin-fixed, paraffin-embedded block of the chin biopsy, as follows: specimens were lysed with proteinase K, then incubated with 0.1-mm silica beads in a thermomixer at 100°C with rapid mixing. DNA was extracted from the lysate with the Genomic DNA Clean & Concentrator 10 kit (Zymo Research, Irvine, CA, USA). We used PCR with 5 µL of the DNA extract and previously described primers (*2*) on a Roche LightCycler 480 (Roche Molecular Systems Inc., Branchburg, NJ, USA) with SYBR Green stain. The PCR target is an ≈400-base-pair portion, including the V3-V4 region, of the 16S ribosomal RNA gene. The amplified product, which we sequenced by using Sanger sequencing, showed identical nucleotides to *M. lepromatosis* strain FJ924 (positions 368–765, GenBank accession no. EU203590) and a 3-nt difference from *Mycobacterium leprae* Br4923 (GenBank accession no. FM211192). 

The presence of *M. lepromatosis* was also confirmed by using *M. lepromatosis*–specific PCR at the National Hansen’s Disease Program (Baton Rouge, LA, USA). In consultation with this program, we prescribed clarithromycin, rifampin, and dapsone in April 2017. Within 3 months of treatment, the patient had decreased skin induration, nasal obstruction, and pinna thickening but minimal improvement in arthralgias or peripheral neuropathy symptoms. No immune reactions occurred during treatment.

Until the advent of molecular methods, all leprosy worldwide was assumed to have been caused by *M. leprae*. In 2008, a novel *Mycobacterium* species, *M. lepromatosis*, was identified by multigene analysis on tissue obtained from 2 immigrants to the United States from Mexico who died from diffuse lepromatous leprosy (*3*), which is endemic to Mexico and Costa Rica and is rarely reported from other geographic locations (*4*).

Comparative genomics show that *M. leprae* and *M. lepromatosis* are closely related and derived from a common ancestor (*4*) with ≈9.1% genetic difference between them, suggesting species-level divergence of ≈10 million (*5*) to 13.9 million (*4*) years ago. A phylogeographic survey suggests that *M. lepromatosis* is not widespread (*4*). Since 2008, PCR testing of patients identified *M. lepromatosis* from Mexico (*1*,*6*), Singapore (*7*), Canada (*8*), and Myanmar and Brazil (*9*).

Unlike *M. leprae* (*10*), *M. lepromatosis* has not been found in armadillos. Red squirrels are infected with *M. lepromatosis* in the United Kingdom (*11*), but the geographic distribution, zoonotic transmission risks, and other animal reservoirs are unknown.

Excluding the patient we report, *M.* *lepromatosis* lepromatous leprosy has been diagnosed in 10 persons (including the 2 index case-patients) in the United States: all were immigrants (8 from Mexico, 2 from Costa Rica) (*3*,*6*,*12*). Our report emphasizes that US citizens can acquire *M. lepromatosis* when traveling to Mexico or other locations as tourists. Our patient’s earliest symptoms of arthritis and neuropathy were in December 2014, suggesting acquisition in Puerto Vallarta (2005) or Acapulco (2007), consistent with the leprosy incubation period of 7–8 years (*6*). His signs and symptoms were similar to those of patients from Mexico (*1*,*6*,*12*,*13*) and a patient from Canada in whom peripheral neuropathy developed 1–2 years before onset of skin lesions. Nasal mucosal involvement was prominent in both. Loss of eyebrows, eyelashes, or both is common in *M. lepromatosis* infection (*6*,*8*,*13*) and might be an early sign of infection. Future reports may help determine if this feature is specific for *M. lepromatosis*. In the case reported by Han et al., (*13*), cure was associated with nearly complete regrowth of eyebrows and eyelashes. 

The full spectrum of manifestations, outcomes, and global burden of *M. lepromatosis* infection remain unknown. In a study of persons who had leprosy, specifically diffuse lepromatous leprosy in Mexico, *M. lepromatosis* was identified more often (63.2%) than *M. leprae* and caused dual infections in 16.1% (*1*).

Leprosy can resemble autoimmune disorders, including systemic lupus erythematosus or rheumatoid arthritis. Chronic polyarthritis is described in up to 75% of leprosy patients, possibly secondary to an immune response to mycobacterial heat-shock proteins (*14*,*15*). Overlapping occurrences of rheumatoid and leprous arthritis make it difficult to differentiate these disorders. The arthritis in the patient we report could be leprous.

Reported patients who have *M. lepromatosis* and *M. leprae* lepromatous leprosy have been treated similarly (*12*). Because of the multibacillary load and higher risk for immune reactions and pigmentation, especially with clofazimine or minocycline, we prescribed clarithromycin, rifampin, and dapsone for this patient. He was maintained, at our advice, on methotrexate to modulate these reactions.

In summary, *M. lepromatosis* lepromatous leprosy is a travel-related hazard for travelers to Mexico or other disease-endemic areas. Specific exposure risks for acquisition of *M. lepromatosis* are unknown. The presence of leprosy-like skin lesions should prompt detailed evaluation, including skin biopsy for histopathology, histochemical stains for mycobacterial organisms, and 16S ribosomal RNA gene PCR to identify the causative agent.
